# Effects of Tart Cherry Juice on Biomarkers of Inflammation and Oxidative Stress in Older Adults

**DOI:** 10.3390/nu11020228

**Published:** 2019-01-22

**Authors:** Sheau C. Chai, Kristina Davis, Zugui Zhang, Longying Zha, Kenneth F. Kirschner

**Affiliations:** 1 Department of Behavioral Health and Nutrition, College of Health Sciences, University of Delaware, Newark, DE 19716, USA; kristinacdavis1@gmail.com; 2 Value Institute, Christiana Care Health System, Newark, DE 19718, USA; zzhang@christianacare.org; 3 Department of Nutrition and Food Hygiene, Guangdong Provincial Key Laboratory of Tropical Disease Research, School of Public Health, Southern Medical University, Guangzhou 510515, China; lyzha@smu.edu.cn; 4 College of Health Sciences, University of Delaware, Newark, DE 19716, USA; kirschne@udel.edu

**Keywords:** cardiovascular risk factors, dietary intervention, inflammation, oxidative stress, polyphenols, tart cherry

## Abstract

Inflammation and oxidative stress are important factors in the development of cardiovascular disease and atherosclerosis. The findings of our previous study suggest that 12 weeks consumption of tart cherry juice lowers the levels of systolic blood pressure (BP) and low-density lipoprotein (LDL) cholesterol in older adults. The present study investigated the effects of tart cherry juice on blood biomarkers of inflammation and oxidative stress. In this randomized-controlled clinical trial, a total of 37 men and women between the ages of 65–80 were randomly assigned to consume 480 mL of tart cherry juice or control drink daily for 12 weeks. Several blood biomarkers of inflammation and oxidative stress were assessed at baseline and after 12 weeks intervention. After the 12 weeks intervention, tart cherry juice significantly increased the plasma levels of DNA repair activity of 8-oxoguanine glycosylase (*p* < 0.0001) and lowered (*p* = 0.03) the mean c-reactive protein (CRP) level compared to the control group. There was a significant group effect observed for plasma CRP (*p* = 0.03) and malondialdehyde (MDA) (*p* = 0.03), and a borderline significant group effect observed for plasma oxidized low-density lipoprotein (OxLDL) (*p* = 0.07). Within group analysis showed that the plasma levels of CRP, MDA, and OxLDL decreased numerically by 25%, 3%, and 11%, respectively after 12 weeks of tart cherry juice consumption compared with corresponding baseline values. The present study suggests that the ability of tart cherry juice to reduce systolic BP and LDL cholesterol, in part, may be due to its anti-oxidative and anti-inflammatory properties. Larger and longer follow-up studies are needed to confirm these findings.

## 1. Introduction

Cardiovascular disease (CVD) continues to be the leading cause of death in the United States. It has been estimated that 80% of people who die of CVD are aged 65 or older. The number of adults who will reach age 65 and older is projected to exceed 82 million by the year 2040, making CVD a major health threat [1]. Atherosclerosis is thought to play a large role in the development of CVD [2]. Atherosclerosis occurs in the innermost arterial wall, called the intima. The intima is comprised of smooth muscle cells lined by endothelial cells. Under normal vascular conditions, monocytes circulate in the blood stream and respond to injury on the arterial wall. In the presence of LDL, the monocytes will slip under blood vessel cells and engulf the LDL molecule, becoming foam cells. The foam cells will develop into thin layers lining the arterial walls, forming fatty streaks. Over time, these fatty streaks will continue to build up and will eventually harden and calcify into atherosclerotic plaque [3]. As the plaque builds up, blood flow will be blocked, leading to high BP. As BP increases, it is possible for the fibrous capping on the plaque to rupture, releasing dangerous thrombus into the blood stream. In addition, as the atherosclerosis process continues and plaque builds up, the arterial lumen will eventually be blocked, leading to ischemic conditions and a cardiovascular event, such as coronary heart disease or stroke [4].

It is known that inflammation and oxidative stress are associated with the atherosclerosis process and impaired vascular endothelial function [5,6,7]. The majority of cells present at the site of plaque rupture are macrophages, T cells, and mast cells, all of which are inflammatory mediators. The macrophages contain metalloproteinases, which break down collagen within the fibrous cap, leaving it more prone to rupture. In addition, the activation of smooth muscle cells present in the fibrous cap will secrete factors that will attract additional monocytes, therefore increasing the inflammatory response and promoting a local pro-coagulant effect, mainly due to the action of proinflammatory cytokines. Furthermore, CRP, a marker of systemic inflammation which is increased in the presence of proinflammatory cytokines, may have an effect on nitric oxide (NO). CRP may reduce the production and bioavailability of NO, leading to a reduction in NO at the site of atherosclerosis. NO is a molecule that inhibits platelet adherence and aggregation and suppresses vasoconstriction. A reduction in NO will further contribute to the inflammatory and atherogenic processes [8]. Reactive oxygen species (ROS) generated by oxidative stress and inflammation are associated with age-related endothelial dysfunction [9]. Endothelial dysfunction is characterized by a shift in the actions of the endothelium toward a proinflammatory state, reduced vasodilation such as NO molecules, and increased vasoconstriction such as endothelial 1 (ET-1) and angiotensin II (ANG-II) molecules [10]. These molecules play a major role in increasing arterial stiffness [11,12,13] and oxidative stress [14], thus causing vascular damage [10].

Many research studies have focused on different diets when it comes to the prevention and treatment of CVD [15,16,17]. Epidemiological studies [18,19,20] also support the existence of an inverse association between a fruit and vegetable-rich diet and CVD, in part, due to their antioxidant content. For instance, according to National Health and Nutrition Examination Survey (NHANES) data, the cumulative mortality from stroke and CVD decreased as fruit and vegetable consumption increased [19]. Findings from the Health Survey from England showed that individuals consuming seven or more servings of fruits and vegetables daily have the lowest risk of all-cause mortality [18]. A recent meta-analysis that included 95 studies found that an increase in fruit and vegetable consumption (0.8 kg/day) resulted in lower risk of CVD and all-cause mortality [21]. The decreased risk of chronic diseases associated with fruit consumption may be attributed to the polyphenolic compounds, fiber, carotenoids, and certain vitamins and minerals such as vitamin C and potassium which are ample in many fruits [13,22,23,24]. Research has shown that polyphenolic compounds may act as antioxidants and interfere with the development of CVD, leading to an inverse relationship between plasma antioxidants and CVD [25]. Montmorency tart cherries (*Prunus cerasus* L.) are a rich source of polyphenolic compounds, especially proanthocyanins, anthocyanins, and flavonols, all of which are strong antioxidants [26,27,28,29,30]. There is ample evidence that tart cherry consumption exerts anti-inflammatory and anti-oxidative properties in vivo and in vitro [7,31,32,33,34]. There is also growing evidence that tart cherries exert cardioprotective effects [31,32,35,36,37,38,39]. Our recently published 12 weeks randomized controlled trial demonstrated that tart cherry juice can lower systolic BP and LDL cholesterol in older adults [40]. As part of our previously published trial, we investigated the effects of tart cherry juice on blood biomarkers of inflammation, and oxidative stress. We hypothesized that the cardioprotective effects of tart cherry juice occur through its anti-inflammatory and anti-oxidative properties.

## 2. Methods

### 2.1. Study Design and Participants

The present study was a 12-week, parallel, randomized controlled trial conducted at the University of Delaware. A detailed description of the study design and participant characteristics have been previously reported [40]. Briefly, men and women between the ages of 65–80 years who consumed ≤ 5 servings of fruits and vegetables per day were recruited via local newspaper advertisements and recruiting materials placed in the local community. After an initial phone screening, potential participants were invited to the study site to complete a screening visit. Medical history, medications/supplement use, and typical food intake were obtained from participants to ensure they met the study criteria. Individuals receiving treatment with any medications that may influence brain function or have had any prior diagnosis or history of stroke, heart disease, diabetes, gastrointestinal disease, cancer, central nervous system or psychiatric disorders, traumatic brain injury, or impaired cognitive function (Montreal Cognitive Assessment (MoCA) score < 19) were excluded. Heavy smokers were also excluded from this study. Based on inclusion and exclusion criteria, a total of 37 participants were enrolled (men = 17; women = 20) in the study. This study was conducted according to the guidelines laid down in the Declaration of Helsinki and all procedures involving human subjects were approved by the Institutional Review Board at the University of Delaware. All participants provided written informed consent before participating in the study. The trial was registered at clinicaltrials.gov as NCT02922920.

### 2.2. Intervention

A total of 37 men and women were randomly assigned to consume either 480 mL tart cherry juice or control drink daily for 12 weeks. A commercially available Montmorency tart cherry concentrate (King Orchards, MI, USA) was used in this study. A detailed description for preparing beverages and nutrient composition of tart cherry juice and control drink were previously reported [40]. Briefly, 68 mL of Montmorency tart cherry concentrate was diluted with 412 mL of water. Control drink was prepared by mixing unsweetened black cherry flavored Kool-Aid (Kraft Foods, Chicago, IL, USA) with water. The control drink was matched for energy and sugar content with similar color, sugar content, acid, and flavor as the tart cherry juice. Participants were instructed to consume 240 mL of the beverage in the morning and 240 mL in the evening for the entirety of the 12-week intervention. 480 mL of tart cherry juice provided 181 kcal of energy, 43 g of carbohydrate, 2.3 g of protein, 8.6 mg of calcium, 46 mg of phosphorous, 355 mg of potassium, 11 of mcg thiamin, 18.6 mcg of folic acid, 6092.8 unit of oxygen radical absorption capacity, 95.9 mg of total tannins, and 450.6 gallic acid equivalents of total phenolics. 480 mL of control drink provided 180 kcal of energy, and 45 g of carbohydrate.

### 2.3. Anthropometric, Dietary and Physical Activity Assessments

Anthropometric, dietary and physical activity assessments were collected at baseline and after 12 weeks intervention. Briefly, a stadiometer was used to measure height to the nearest 0.1 cm. Weight was measured in kilograms with minimal clothing on a digital scale to the nearest ± 0.1 kg. Body mass index (BMI, kg/m^2^) was calculated. Three-day food records were collected and analyzed using Nutrition Data System for Research software (NDSR, Minneapolis, MN, USA).

### 2.4. Blood Collection

Fasting venous blood for plasma was collected after an overnight fast at baseline and after 12-week intervention. The plasma was separated by centrifuging at 1500 g for 15 minutes at 4 °C within 1 h using a Beckman Allegra 6KR centrifuge (Beckman Coulter, Inc., Brea, CA, USA). Samples were then aliquoted and stored at −80 °C until analyses.

### 2.5. Blood Biomarkers

Inflammatory and oxidative stress biomarkers were assessed at baseline and after 12-week intervention. Plasma CRP, tumor necrosis factor-alpha (TNF-α), NO, and ET-1 were measured using ELISA kits from R&D System Inc. (Minneapolis, MN, USA). Plasma 8-hydroxydeoxyguanosine (8-OHdG) and MDA were assessed using ELISA kit from Cell Biolabs, Inc. (San Diego, CA, USA). Plasma 8-oxoguanine glycosylase (OGG1) and OxLDL were assessed using ELISA kit from LifeSpan BioSciences, Inc. (Seattle, WA, USA). Plasma 4-hydroxynonenal (4HNE) was assessed using ELISA kit from Elabscience Biotechnology (Bethesda, MD, USA). Plasma ANG II was assessed using ELISA kit from RayBio (Norcross, GA, USA).

### 2.6. Statistical Analysis

All analyses were performed using SAS 9.3 (SAS Institute Inc., Cary, NC, USA). The Wilcoxon rank sum test was used to compare continuous variables between two groups, including the mean changes in outcome variables during the intervention period. Contingency table analysis (chi-square or Fisher exact test) were used to compare categorical variables. To account for the potential effect of tart cherry juice on final outcome variables, the 12-week outcomes were estimated via ANCOVA analysis, with adjustment for physical activity, dietary cholesterol, and MoCA which showed significant baseline differences between tart cherry and control group. The final adjusted outcome variables were compared and utilized to examine the impact of group, time, and the interaction of group and time via mixed ANOVA. Values are reported as mean ± standard deviation (SD). In all statistical comparisons, differences with *p* < 0.05 were considered significant.

Sample size calculation was based on Traustadottir et al. [7]. A sample size estimate of 36 (18 in each group) would allow for an attrition rate of 15% and still maintain power of 0.80, with *α* = 0.05 and *r* = 0.30 for detecting a moderately small effect (Cohen’s *f* = 0.31) in 8-oxoguanine, an oxidative stress biomarker.

## 3. Results

### 3.1. Baseline Characteristics, Anthropometric Measurements, Dietary Intake and Physical Activity

As previously reported [40], 34 participants successfully completed the study. There were no significant differences between tart cherry and control groups for baseline age (69.5 ± 3.9 years in control; 70.0 ± 3.7 years in tart cherry; *p* = 0.65), height (169.6 ± 7.9 cm in control; 165.5 ± 6.8 cm in tart cherry; *p* = 0.25), body weight (78.7 ± 13.5 kg in control; 78.0 ± 10.3 kg in tart cherry; *p* = 0.56), BMI (27.3 ± 4.2 in control; 28.5 ± 3.7 in tart cherry; *p* = 0.34) and sex (9 males and 8 females in control; 8 males and 12 females in tart cherry; *p* = 0.43) except for MoCA (26.7 ± 2.1 in control; 24.8 ± 2.5 in tart cherry; *p* = 0.02). After 12 weeks intervention, there were no significant changes in body weight in both groups. The BMIs were significantly different between the two groups (mean difference of 1.06; 95% CI: 0.18 to 1.94; *p* = 0.02) without significant impacts of time, group, and interaction of time and group. The baseline mean physical activity (135.8 ± 37.3 in control; 224.5 ± 190.6 in tart cherry; *p* < 0.05) was higher and the baseline mean dietary cholesterol (262.0 ± 133.9 mg in control; 203.0 ± 90.3 mg in tart cherry; *p* < 0.05) was significantly lower in the tart cherry group than in the control group, respectively. In the present study, participants were asked to maintain their habitual diet and physical activity levels during the course of the study. However, the within group analysis showed that physical activity level significantly decreased in the tart cherry group (*p* < 0.0001) and control group (*p* < 0.0001) while dietary cholesterol significantly increased in the tart cherry group (*p* < 0.001) and control group (*p* = 0.04).

### 3.2. Biomarkers of Inflammation, Oxidative Stress, and Vascular Function

Blood biomarkers of inflammation and oxidative stress are presented in Table 1. Baseline levels of OGG1, CRP, and OxLDL were not statistically different between tart cherry and control groups. Between-group analysis indicated that after the 12 weeks intervention, participants in the tart cherry group had higher levels of the DNA repair activity of OGG1 (mean difference of 31.2; 95% CI: 18.3 to 44.1; *p* < 0.0001) than in the control group. There was a significant group effect observed for plasma CRP (*p* = 0.03) and a borderline significant group effect observed for plasma OxLDL (*p* = 0.07). Between-group analysis indicated that the CRP levels were significanty different between two groups (mean difference of 0.44; 95% CI: 0.06 to 0.82; *p* = 0.03) after 12 weeks intervention. Within group analysis showed that tart cherry juice consumption numerically decreased CRP levels by 25% and OxLDL by 11% at 12 weeks compared with corresponding baseline values.

Similarly, there was a significant group effect observed for MDA (*p* = 0.0025). Between-group analysis indicates that MDA levels were significantly different between the two groups (mean difference of 0.30; 95% CI: 0.09 to 0.51; *p* = 0.01) after 12 weeks intervention. Of note, the baseline levels of MDA were different between the two groups (*p* = 0.04). Within group analysis showed that tart cherry juice consumption numerically decreased MDA level by 3% compared with baseline.

Baseline levels of TNF-α, 4HNE, 8-OHdG, ANG-II, ET-1, and NO were not statistically different between tart cherry and control groups. There were no significant changes in TNF-α, 4HNE, 8-OHdG, ANG-II, ET-1, and NO as a result of tart cherry juice or control drink consumption. In addition, there were no significant group, time, or group x time interaction effects observed for these biomarkers.

## 4. Discussion

In our earlier study [40], we found that there was a significant group × time interaction effects for systolic BP and LDL cholesterol. After the 12 weeks intervention, older adults in the tart cherry group had lower systolic BP and LDL cholesterol compared to control group. To understand the underlying mechanisms through which tart cherry juice reduces systolic BP and LDL cholesterol, several blood biomarkers of inflammation and oxidative stress were assessed in the present study. To our knowledge, the present study is the longest-term human trial that has examined the health protective effects of tart cherry consumption. Our current study demonstrated that tart cherry juice significantly increased the DNA repair activity of OGG1 and lowered the levels of CRP compared to the control group after the 12 weeks intervention. In addition, there was a significant group effect observed for CRP and MDA, and a borderline significant group effect observed for OxLDL. The plasma levels of CRP, MDA, and OxLDL decreased numerically by 25%, 3%, and 11%, respectively, after 12 weeks of tart cherry juice consumption compared with corresponding baseline values. These reductions are of important clinical relevance with respect to natural ways of improving inflammatory and oxidative stress status. Other biomarkers including TNF-α, 4HNE, 8-OHdG, ANG-II, ET-1, and NO were not affected by tart cherry juice or control consumption. It is important to note that the final outcome variables of this study were adjusted for baseline physical activity, dietary cholesterol, and MoCA score. Adjusting for the covariates would provide a more precise effect estimate. It is noteworthy that our results remained significant after adjusting for baseline covariates.

Inflammation and oxidative stress are associated with the development of CVD. Oxidative stress is an “imbalance between the rate of formation and the rate of clearance of ROS” and may play a key role in the development of atherosclerosis [7,41]. The fundamental mechanisms by which ROS contribute to atherogenesis are through oxidation of LDL cholesterol, endothelial dysfunction, vascular smooth muscle cell growth, and monocyte migration [42,43]. Oxidized lipids may be important mediators of lipid peroxidation and are known to initiate foam cell formation and accelerate plaque formation [44,45]. Collectively, our findings support the notion that the cardioprotective effects of tart cherry juice, in part, may be due to its anti-inflammatory and anti-oxidative properties, as it reduces CRP levels, and increases OGG1 levels over the 12 weeks in older adults.

Aside from our present findings, there is ample evidence that tart cherry juice consumption provides anti-inflammatory and anti-oxidative properties in vivo and in vitro [7,31,32,33,34]. But they employed different study designs, populations/animal models, and dosages of tart cherry concentrate. In a crossover study by Traustadottir et al. [7], older adults were ischemic reperfusion and randomly assigned to consume 240 ml of tart cherry or a placebo juice twice daily for 14 days. This study showed that older adults who consumed tart cherry concentrate for 14 days had lower urinary levels of 8-hydroxy-2′-deoxyguanosine and 8-hydroxyguanosine, biomarkers of oxidative stress. Another human study by Schumacher showed that 480 ml tart cherry juice consumption for 6 weeks significantly decreased CRP in adults with mild to moderate osteoarthritis [34], which supports our finding. Another crossover study by Martin et al. [46] showed that 240 ml tart cherry juice consumption for 4 weeks significantly decreased proinflammatory monocyte chemoattractant protein 1 (MCP-1) in overweigh and obese adults compared to the placebo group. However, CRP levels were not affected by tart cherry juice, which is inconsistent with our finding. An animal study by Saric et al. [32] demonstrated that mice that were fed tart cherries for 14 days showed evidence of increased superoxide dismutase activity in both the liver and the blood. In addition, the mice showed increased liver glutathione peroxidase, reduced lipid peroxidation, and decreased cyclooxygenase (COX)-II activity. A study by Mulabagal et al. [31] also showed that tart cherries were able to inhibit lipid peroxidation and COX enzyme activity. Seymour et al. [39] using the Zucker rat model, rats that were fed a high-fat diet, supplemented with 1% (wt/wt) freeze-dried whole tart cherry powder for 90 days, were found to reduce interleukin (IL)-6 and TNF-α levels. A recent animal study by Jayarathne et al. [47] showed that Zucker fatty rats that were fed 4% tart cherry powder diet for 8 weeks showed evidence of reduced mRNA levels of pro-inflammatory markers including IL-6, TNF-α, IL-1β, MCP-1, inducible nitric oxide synthase, and CD-11b, and increased mRNA levels of type-1 arginase.

Consumption of fruits containing various polyphenolic compounds decrease CVD prevalence. Tart cherries are a particularly rich source of anthocyanins, specifically cyanidin-3-glucosylrutinoside, cyanidin-3-rutinoside, cyanindin-3-glucoside, and their agylcone, cyaniding [28,48]. It has been proposed that the mechanism behind tart cherry’s health benefits is related to the bioactive compounds present in tart cherries, including various polyphenolic compounds that act as antioxidants. Compared to many foods that are rich in antioxidants, the level of antioxidant activity in tart cherries is similar to blueberries, strawberries and pomegranates [33]. Tart cherries have higher amounts of antioxidants compared to many other foods per portion size than red wine, dark chocolate, and orange juice, placing tart cherry at rank 14 out of the top 50 highest antioxidant containing foods [49]. An analysis of polyphenol content was conducted by Ou et al. [33] to evaluate how processing may affect the polyphenolic and antioxidant properties in tart cherry juice concentrate, dried, frozen and canned tart cherries. In nearly all measures of antioxidant activity, including oxygen radical absorbance capacity and several other measures including hydroxyl, peroxyl, and super oxide radical absorbance capacities, tart cherry juice concentrate had higher values than the other processed cherries, with the exception of super oxide radical absorbance capacity, which had a value that was slightly less than that of dried cherries. Ou et al. [33] also found that tart cherry concentrate had higher inhibitory actions than did other types of processed tart cherries. Seeram et al. [29] found that the aglycone cyanidin from tart cherries showed comparable anti-inflammatory activities to ibuprofen and the non-steroidal anti-inflammatory drug naproxen when comparing COX-I and COX-II inhibition activities.

With respect to the influence of tart cherry on body weight, BMI, glucose and insulin sensitivity, our findings suggest that daily incorporation of tart cherry juice or control drink into a diet do not significantly affect the total energy, carbohydrate, fat and protein intakes. As such, we did not observe a significant increase in body weight in the current study. Although the BMIs and glucose levels were different between the two groups after 12 weeks intervention, there were no group, time, or group × time interaction effects observed for BMI, insulin and insulin resistance index (HOMA-IR). Twelve weeks supplementation with tart cherry juice or control drink did not affect insulin and HOMA-IR levels [40]. Sugar-sweetened beverages are commonly consumed by Americans. According to NHANES data [50], adults are consuming approximately 209 kcal per day from sugar-sweetened beverages. 480 ml of tart cherry juice provides an approximately 181 kcal of energy and 34 g of sugar. These amounts of energy and sugar are lower than other commonly consumed fruit juices and soft drinks in the United States [51]. The findings from our 3-day food records also showed that the majority of the participants in the present study replaced their average daily sugar-sweetened drink with tart cherry juice or a control drink. The compliance to the supplement in those participants who completed the study was on average 94%. This suggests that tart cherry juice can easily be incorporated into Americans’ daily diet.

## 5. Conclusions

In summary, our research findings suggest that regular tart cherry juice consumption helps to reduce CVD risk factors in older adults by reducing systolic BP and LDL cholesterol [40]. The BP- and LDL-lowering effect of tart cherry juice may be through its anti-oxidative and anti-inflammatory properties, as evidenced by an increase in the plasma levels of DNA repair activity of OGG1 with a reduction in CRP levels compared to the control. This study reinforces the notion that consuming fruits and vegetables, or their derived products, can have significant benefits on the CVD risk profile. Age is an independent risk factor of CVD. Inflammation and oxidative stress increase with advancing age are associated with the development of chronic disease. Our findings warrant further investigation in a larger sample size and longer-term trial in an older population.

## Figures and Tables

**Table 1:** Blood biomarkers of inflammation and oxidative stress in older adults at baseline and 12 weeks after supplementation with tart cherry juice or control drink.

			^a^ Difference between Two Groups			^a^*p*-Value (ANOVA)		
Variables	Control (*n* = 17)	Tart Cherry (*n* = 17)	Difference	95% CI	*p*	Group	Time	Interaction
TNF-α, pg/mL								
Baseline	1.58 ± 1.53	1.13 ± 1.28	−0.20	−0.49, 0.10	0.19	0.1956	0.6762	0.4430
12 weeks	1.49 ± 1.18	1.30 ± 0.34						
CRP, mg/mL								
Baseline	1.52 ± 0.81	3.11 ± 3.20	0.44	0.06, 0.82	0.03	0.0338	0.9176	0.1968
12 weeks	1.89 ± 0.52	2.34 ± 1.13						
4HNE, ng/mL								
Baseline	13.33 ± 1.81	14.34 ± 4.46	1.14	−0.30, 2.59	0.12	0.1188	0.8927	0.9247
12 weeks	13.36 ± 1.35	14.50 ± 2.46						
MDA, pmole/L								
* Baseline	2.89 ± 0.57	3.30 ± 0.58	0.30	0.09, 0.51	0.01	0.0025	0.7781	0.6463
12 weeks	2.91 ± 0.36	3.21 ± 0.22						
OGG1, ng/mL								
Baseline	321.5 ± 125.5	298.7 ± 96.4	31.2	18.3, 44.1	<0.0001	0.8316	0.5795	0.1764
12 weeks	283.5 ± 23.0	314.7 ± 11.0						
8-OHdG, ng/mL								
Baseline	14.97 ± 4.08	15.41 ± 5.95	0.19	−1.64, 2.01	0.83	0.7499	0.2512	0.8959
12 weeks	13.96 ± 1.76	14.14 ± 3.09						
ANG-II, pg/mL								
Baseline	48.22 ± 35.00	49.89 ± 21.07	−0.32	−7.90, 7.26	0.93	0.8891	0.7105	0.8605
12 weeks	47.31 ± 11.72	46.99 ± 9.57						
ET-1, pg/mL								
Baseline	1.84 ± 0.61	1.78 ± 0.55	−0.09	−0.26, 0.07	0.25	0.4614	0.5415	0.8848
12 weeks	1.92 ± 0.29	1.83 ± 0.15						
NO, umol/L								
Baseline	16.20 ± 11.99	11.48 ± 6.68	−0.76	−6.09, 4.57	0.77	0.1644	0.6113	0.2957
12 weeks	15.49 ± 2.86	14.72 ± 9.72						
OxLDL, pg/mL								
Baseline	0.29 ± 0.14	0.38 ± 0.15	0.02	−0.03, 0.06	0.44	0.0742	0.8274	0.2306
12 weeks	0.32 ± 0.02	0.34 ± 0.08						

Values are means ± standard deviation (SD). ANG-II, angiotensin II; CRP, C-reactive protein; ET-1, endothelial 1; MDA, malondialdehyde; NO, nitric oxide; OGG1, 8-oxoguanine glycosylase; OxLDL, oxidized low-density lipoprotein; TNF-α, tumor necrosis factor alpha; 4HNE, 4-hydroxynonenal; 8-OHdG, 8-hydroxydeoxyguanosine. ^a^ The difference in each group was obtained via the outcome between baseline and 12-week, which was adjusted for baseline physical activity, dietary cholesterol, and MoCA score, assessed by ANCOVA; the difference between two groups was then evaluated via the Wilcoxon rank sum test, to account for the possibility of non-normal distribution. ^a^ *p*-value for the effects of group, time, and interaction of group and time via ANOVA. * *p* < 0.05 for baseline differences.
